# Influence of convective heat transfer coefficients on thermal behaviour of a lithium-ion cell: a numerical study

**DOI:** 10.55730/1300-0527.3645

**Published:** 2023-10-29

**Authors:** Uğur MORALI

**Affiliations:** Department of Chemical Engineering, Eskişehir Osmangazi University, Eskişehir, Turkiye

**Keywords:** Energy storage, lithium-ion battery, convective heat transfer coefficient, battery temperature

## Abstract

In the current study, the impact of C-ratio, convective heat transfer coefficient, and free stream temperature on the maximal cell temperature and temperature uniformity was computationally and statistically examined. Results revealed that the free stream temperature was the main influential factor for the maximal cell temperature for both natural and forced convection conditions while the C-ratio was the most effective parameter for the temperature uniformity for both natural and forced convections. On the other hand, the contribution of the free stream temperature to the maximum battery temperature increased from 63% to 94% when the conditions were changed from natural convection to forced convection. Moreover, the contribution of the C-rate to the temperature uniformity decreased from 89% to 79% when the conditions were changed from natural convection to forced convection. The results obtained from this study are significant in terms of determining which factor should be given more importance under natural and forced convection conditions.

## 1. Introduction

In recent years, the focus on environmentally friendly vehicles has increased, particularly hybrid electric vehicles and electric vehicles [1]. This feature of electric vehicles will play an important role in eliminating many problems, especially global warming [2]. In electric vehicles, technologically advanced energy storage devices are used instead of traditional fossil fuels [3]. In recent years, lithium-ion batteries (LIBs) have come to the fore among energy storage devices [4]. It should be noted that lithium-ion batteries have the potential to generate heat while in use [5]. In excessive operating conditions such as high discharge rates, the amount of heat released increases significantly [6]. It can be stated that high temperatures may have a negative impact on the performance of LIB cells. Therefore, the heat released during the discharge of LIB cells should be removed [7].

There are many articles in the literature examining the thermal behaviour of LIBs. Cooling systems such as air cooling [8,9], liquid cooling [10,11], and phase change material cooling [12,13] have been studied to improve the thermal performance of LIB cells. Hai et al. analysed the impact of air inlet velocity on the thermal behavior of the LIB cell pack [14]. The temperature of the batteries with aligned and nonaligned arrangements was obtained by simulations. It was found that the cell package temperature and outlet air temperature decreased with the increment in the air flow rate. Moreover, the results revealed that the aligned battery pack exhibits better thermal behavior. Hasan et al. designed a system based on air cooling for enhancing the thermal behaviour of LIBs [15]. The result indicated that the average air temperature decreased and the temperature distribution improved with the increase in Reynolds number. In addition, the results displayed that the average heat transfer rate (Nusselt number) of the cell pack increased with the increment in the air inlet velocity (Reynolds number). In another study, Chen et al. achieved a nifty solution for cooling a prismatic cell module: a parallel liquid cooling system [16]. The results indicated that the most effective factor on the cooling effect, energy cost, and temperature uniformity was the mini channel depths. The result demonstrated that the volume energy density and the energy cost were increased by 9% and 27%, respectively. Xu et al. studied the impact of C-ratio, inlet temperature, and flowrate on the thermal behaviour of LIBs for distinct types of liquid cooling approaches [17]. The results indicated that an effective cooling performance was obtained for the F-2 type liquid cooling system. It was discovered that in order to properly cool batteries during discharge at a rate of 1C, the flow rate of the cooling liquid should exceed 6 L/h. Similarly, for a discharge rate of 2C, the flow rate should be greater than 12 L/h. Li et al. developed a composite phase change material by incorporating an electrically insulating material [18]. The result revealed that the composite phase change material exhibited better thermal properties than other structures. The result also indicated that the composite phase change material enhanced the cooling capacity of the cell module compared to natural cooling. Zhou et al. examined the influence of various phase change material parameters such as phase change temperature, structure, and thickness on the cooling capacity of prismatic lithium-ion cells [19]. The result demonstrated that decreasing the phase change temperature and increasing thickness enhanced the cooling capacity of the battery system. Moreover, it was determined that the phase change thickness of 25 mm was optimal thickness considering the temperature uniformity.

There are three multiscale multidimensional (MSMD) models namely Newman, Tiedemann, Gu, and Kim (NTGK), pseudo 2 dimensional (P2D), and equivalent circuit model (ECM) in ANSYS Fluent. In addition to the studies summarized above, there are many studies in the literature in which the thermal behavior of LIB cells is studied numerically. According to Zhang et al., the NTGK model can be utilized to analyse the thermal performance of lithium-ion battery packs [20]. The designed cooling system based on water cooling indicated that the cell temperature and temperature uniformity can be ensured at a tolerable level by adjusting the temperature and inlet velocity at a 5C discharge rate. Kök and Alkaya analyzed the discharge operation of the LIBs by using the NTGK model to investigate the thermal performance [21]. It was determined that the cell temperature increased by increasing the discharge rate from 0.5C to 3C. Moreover, heterogeneous temperature distribution was observed under high discharge rates. Celik et al. examined the thermal behavior of cylindrical LIB cells under distinct discharge conditions by the NTGK model [22]. The effects of free stream temperature, C-ratio, and convective heat transfer coefficient (HTC) on the cell temperature were obtained. As the discharge rate increased, the study found that the convective HTC had a greater impact. Furthermore, it has been found that increasing the convective HTC is necessary to avoid battery overheating during high discharge rates. Paccha-Herrera et al. conducted a comparison of thermal models for a 26650 lithium-ion battery composed of LiCoO_2_ [23]. The results revealed that the NTGK model has a low error rate at low discharge rates and a higher error rate at high discharge rates. All models exhibited the same amount of error for the driving cycle, and the lumped model was found to be more suitable for implementation under wide operating conditions. In a recent study, Kumar et al. used a single particle model to obtain the charge-discharge behavior of a prismatic LIB under distinct C-rates [24]. The discharge profiles obtained with the single particle model were used to obtain the thermal behaviors with the NTGK model at 0.5, 1, and 2 C rates. The results showed that the errors between the experimental studies and the simulation results were less than 1%.

Although there have been a number of studies in the literature, there is nonetheless room for the prediction of the thermal behavior of lithium-ion cells by the NTGK method. In this study, the NTGK model was applied to evaluate the thermal behaviour of LIB cells under distinct convection conditions. Furthermore, the impacts of free stream temperature, C-ratio, and convective HTC on maximal cell temperature and temperature uniformity were determined quantitatively.

## 2. Materials and methods

### 2.1. Materials

**Figure 1:**
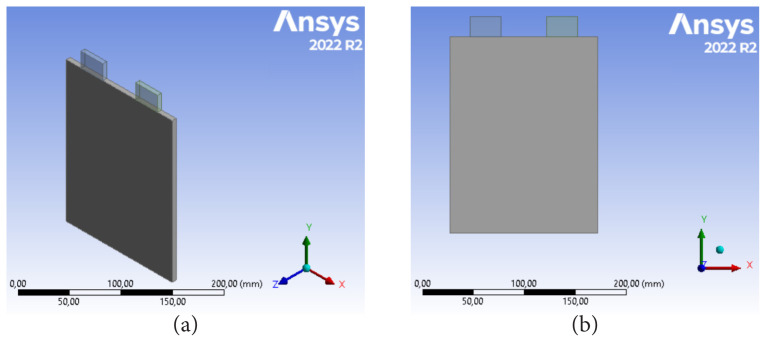
Geometry of lithium-ion cell.

A publicly available LiMn_2_O_4_/graphite prismatic battery was utilized for the simulations. The available potential interval of the Li-ion batteries is between 3.00 V (0% SOC) and 4.20 V (100% SOC). The nominal capacity was 14.6 Ah. Table 1 presents LiMn^2^O^4^ - 14.6 Ah lithium-ion prismatic cell specifications. Figure 1 shows the general geometry of LIB cell. Thermophysical features of LIB cells and tab materials are presented in Table 2.

### 2.2. Methods

#### 2.2.1. Governing equations and NTGK modeling

Valuable information in analyzing battery thermal behavior can be obtained by battery thermal simulations. Concerning the NTGK model, input factors and coefficients can be obtained by discharge data. The NTGK model parameters obtained in [25] were implemented in the current work. The heat generated by a LIB cell during operation is represented in Equation 1 [26,27].


(1) $$\frac{\partial \rho c_{p}T}{\partial t}=\sigma_{+}{|\nabla \phi_{+}|}^{2}+\sigma_{-}{|\nabla \phi_{-}|}^{2}+Q_{g}$$

(2) $$\nabla (\sigma_{+}\nabla \phi_{+})=(j_{short}-j_{ECh})$$

(3) $$\nabla (\sigma_{-}\nabla \phi_{-})=j_{ECh}-j_{short}$$

(4) $$j_{ECh}=\alpha Y[U-(\phi_{+}-\phi_{-})]$$

where *C_p_* and *ρ* present specific heat and density of the battery. *σ_+_* and *σ_−_* represent the effectual conductivity. *ϕ_+_* and *ϕ_−_* represent the phase potentials. *Q_g_* is the heat generated by the battery. *j_ECh_* and *j_short_* are the volumetrical current transference rate and the current transference rate, respectively. There is a functional relationship between the *U* and *Y* coefficients and the depth of discharge (*DoD*) [14,15]


(5) $$U=x_{0}+{\text{x}}_{1}(DoD)+{\text{x}}_{2}{\left(DoD\right)}^{2}+{\text{x}}_{3}{\left(DoD\right)}^{3}$$

(6) $$Y={\text{x}}_{4}+{\text{x}}_{5}(DoD)+{\text{x}}_{6}{\left(DoD\right)}^{2}$$

where x*_0_*-x*_6_* are the fitting parameters. *Y* and *U* coefficients implemented in our work were received from the work of [15]. The depth of discharge *(DoD)* is described as:


(7) $$Depth\;of\;discharge=\frac{\int_{0}^{t}Jdt}{Q_{T}}$$

where *J* is the current intensity dispersion, *t* shows the time elapsed during the charge/discharge process, and *Q_T_* is the electrode capacity (Ah/m^2^).

#### 2.2.2. Mesh independence

**Figure 2:**
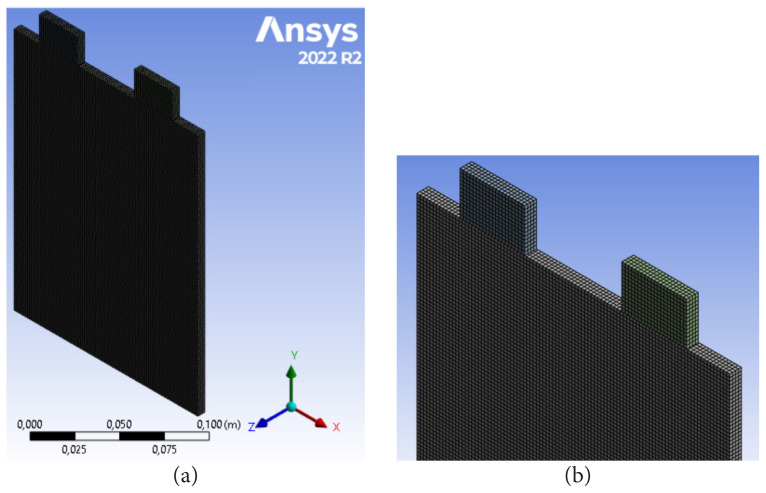
Mesh structure of LIB cell.

**Figure 3:**
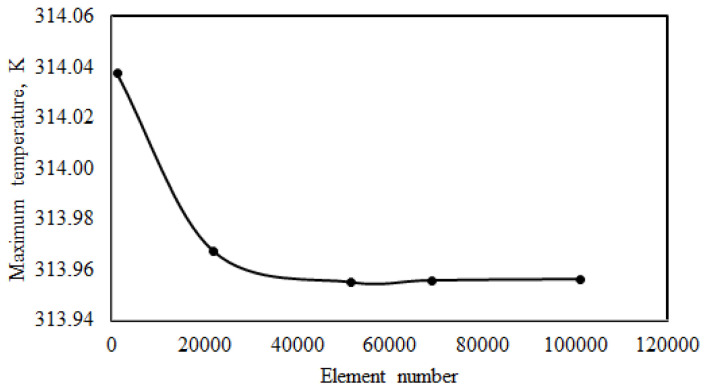
Mesh independence of lithium-ion battery.

The results obtained with the simulation are highly dependent on the mesh structure. Therefore, a mesh independence test should be applied before the simulations are run. Therefore, the relationship between different mesh numbers and the maximal battery temperature was determined at a 4C discharge ratio. The convective heat transfer coefficient and free stream temperature were 5 W/mK and 300 K, respectively. The mesh structure of the LIB cell is depicted in Figure 2. The results of mesh independence are presented in Figure 3. Figure 3 revealed that the maximum cell temperature first decreased and then nearly remained constant as the number of meshes increased. The maximal battery temperature changed from 313.9549 K to 313.9556 K as the mesh count rose from 51,744 to 69,064. The amount of change was less than 0.1%. For this reason, the mesh number 51,744 was chosen for further studies in order to ensure that the result is independent of the mesh number and to get faster results.

#### 2.2.3. Experimental validation

**Figure 4:**
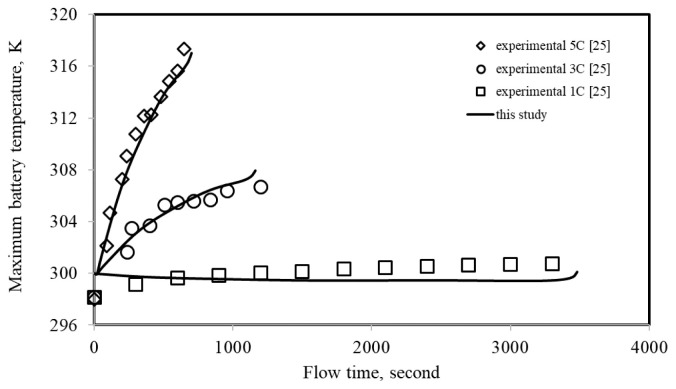
Relationship between the simulations and experimental result.

It is expected that the results obtained with the simulation will be in agreement with the experimental result. Therefore, a confirmation test was performed at different discharge rates, including 1C, 3C, and 5C. Free stream temperature and HTC were 300 K and 5 W/mK, respectively. Results shown in Figure 4 revealed that the simulation outcomes matched well with the experiential findings.

#### 2.2.4. Statistical analysis

In this study, the effects of free stream temperature (FST), convective heat transfer coefficient (HTC), and C-ratio on maximal cell temperature and temperature uniformity were investigated by the Taguchi experiment design method. Experimental design not only allows simulations to be performed with a small number of performances but also allows statistical evaluation of the results. In other words, the results that could not be obtained as a result of the examinations made with the traditional methods were obtained by using the Taguchi experimental design method. Determining the objective function is the 1st step of the Taguchi method. The objective functions in the current work are maximal battery temperature and temperature uniformity. In this work, while it was desired to minimize the maximum cell temperature by controlling various factors, a homogeneous temperature distribution was also expected. The 2nd stage of the Taguchi design is to determine the determinants that can influence the objective function. Controllable factors in this study were selected as FST, C-rate, and HTC. Controllable factors and their levels are shown for natural convection and forced convection in Table 3. The third step in Taguchi’s experimental design is the determination of the orthogonal array. The L9(3^3^) orthogonal arrangement is shown in Tables 4 and Table 5 for natural and forced convection, respectively. The fourth step of the Taguchi design is to run simulations and collect data. In this step, simulations were performed according to the conditions given by the orthogonal array. Minimal cell temperature and maximal cell temperature values were obtained with simulations. Temperature uniformity was determined by calculating the difference between the maximal cell temperature and the minimum cell temperature. The final step of the Taguchi design is the analysis of the collected data. The analysis process was carried out using the smaller-is-better characteristic since it was desired that the maximal cell temperature and temperature uniformity functions be minimal.

## 3. Results and discussions

The temperature results for both natural convection and forced convection were presented as functions of the FST, the C-ratio, and the HTC. Then the results were discussed considering the Taguchi design.

Results of battery temperatures (maximal cell temperature and minimal cell temperature) for natural convection and forced convection are tabulated in Table 6 and Table 7, respectively. Temperature uniformity (dT) was reflected by the distinction between the maximal battery temperature and the minimal battery temperature. Concerning the natural convection conditions, the biggest maximal cell temperature of 317.7783 K was estimated for n9 while the biggest minimum cell temperature of 316.7337 K was also estimated for n9. On the other hand, for the natural convection conditions, the largest temperature uniformity value (1.1203) was calculated for n8. Concerning the forced convection conditions, the highest maximal battery temperature of 313.5813 K was obtained for f9 while the highest minimum cell temperature of 313.3024 K was also estimated for f9. Similarly, for the forced convection conditions, the largest temperature uniformity value (0.3317) was calculated for f8. The S/N ratios for the maximal cell temperature and dT are also shown in Table 6 and Table 7 for the natural and forced convection conditions, respectively.

Table 8 shows the response Table for the maximal cell temperature for natural convection. As can be seen from Table 8, the FST factor had the highest delta value with a value of 0.1368, indicating the highest impact of the FST on the maximal cell temperature. The delta (Δ) value of the C-ratio was 0.0604 which was lower than the HTC. The HTC exhibited the lowest delta value of 0.02. Additionally, Table 8 showed that the FST’s contribution to the maximal cell temperature was 63%. For natural convection, the contribution of C-rate and HTC to the maximum battery temperature was 28% and 9%, respectively. Table 9 shows the response Table for the maximal cell temperature for forced convection. The highest delta value for the forced convection conditions was obtained for the FST factor with a value of 0.1408 for the maximal cell temperature. The highest Δ value of the FST demonstrated that concerning the forced convection conditions the impact of the FTS on the maximal cell temperature was the highest. The Δ value of the C ratio and the HTC factors was 0.0070 and 0.0018, respectively. This indicated that the impact of the C-ratio on the maximal cell temperature was larger than that of the HTC for forced convection conditions. Additionally, Table 9 showed that the FST’s contribution to the maximal cell temperature was 94%. For forced convection, the contribution of C-rate and HTC to the maximum battery temperature was 5% and 1%, respectively.

Table 10 shows the response Table for the temperature uniformity for natural convection. The largest Δ value of 8.5512 was obtained for the C-ratio. The Δ value of the HTC (0.9083) was lower than that of the FST (0.1976). These results revealed that the C-ratio was the most powerful factor in the temperature uniformity for natural convection conditions. Moreover, concerning the natural convection conditions the influence of the HTC on the temperature uniformity was higher than that of the FST. Additionally, Table 10 presented that the contribution of the C-ratio to the temperature uniformity was 89% for the natural convection conditions. Furthermore, the contribution of the HTC and FST to the temperature uniformity was 9% and 2%, respectively. Table 11 shows the response Table for the temperature uniformity for forced convection. According to Table 11, the delta values of the factors were 6.4167, 0.9875, and 0.6878 for the C-rate, HTC, and FST, respectively. The largest Δ value calculated for the C-ratio revealed the biggest impact of the C-ratio on the temperature uniformity for forced convection conditions. Moreover, the impact of the HTC on the temperature uniformity was higher than that of the FST for forced convection conditions. Furthermore, Table 11 indicated that the contribution of the C-ratio to the temperature uniformity was 79% for the forced convection conditions. Additionally, the contribution of the HTC and FST to the temperature uniformity was 12% and 9% for the forced convection conditions, respectively.

## 4. Conclusion

An L9 (33) orthogonal array was employed to assess the impacts of the C-ratio, heat transfer coefficient, and free stream temperature on maximal cell temperature and temperature uniformity. The primary results that can be drawn from the current study are listed below:

- The control factor with the most important impact on the maximal cell temperature was free stream temperature with a contribution of 63% and 94% for natural and forced convections, respectively.
- The control factor with the most significant influence on the temperature uniformity was C-rate with a contribution of 89% and 79% for natural and forced convection conditions, respectively.
- The heat transfer coefficient has the least notable influence on the maximum cell temperature. Moreover, less attention can be paid due to the limited contribution to the free stream temperature for temperature uniformity.
- The results obtained can offer quantitative information to assist in the creation of thermal management systems for LIB cells.

## Figures and Tables

**Table 1:** Physical parameters of lithium-ion cell.

Parameter	Value
Positive electrode	LiMn_2_O_4_
Negative electrode	Graphite
Electrolyte	Polymer-based
Length	19.2 cm
Width	14.5 cm
Thickness	0.54 cm
Nominal capacity	14.6 Ah
Charge cut-off potential	4.2 V
Discharge cut-off potential	3.0 V

**Table 2:** Thermal and physical properties of lithium-ion cell.

Property	Battery	Negative tab	Positive tab
Density, kg m^−3^	2092	8030	2719
Specific heat, J kg^−1^ K^−1^	678	502.48	871
Thermal conductivity, W m^−1^ K^−1^	18.2	16.27	202.4
Electrical conductivity, S m^−1^	-	8.330 × 10^6^	3.541 × 10^7^

**Table 3:** Levels of factors for natural convection and forced convection.

	C-rate, h^−1^	FST, °C	HTC, W m^−1^ K^−1^	
			Natural convection	Forced convection
1^st^ Level	1	30	5	150
2^nd^ Level	2	35	10	200
3^rd^ Level	3	40	15	250

**Table 4:** Orthogonal matrix for natural convection.

Run	C-rate	FST	HTC
n1	1	30	5
n2	1	35	10
n3	1	40	15
n4	2	30	10
n5	2	35	15
n6	2	40	5
n7	3	30	15
n8	3	35	5
n9	3	40	10

**Table 5:** Orthogonal matrix for forced convection.

Run	C-rate	FST	HTC
f1	1	30	150
f2	1	35	200
f3	1	40	250
f4	2	30	200
f5	2	35	250
f6	2	40	150
f7	3	30	250
f8	3	35	150
f9	3	40	200

**Table 6:** Results of battery temperatures for natural convection.

Run	Tmax	Tmin	dT	Tmax_SN	dT_SN
n1	304.6165	304.4503	0.1662	−24.8375	7.7927
n2	308.9460	308.8024	0.1435	−24.8988	8.4300
n3	313.6768	313.5519	0.1249	−24.9648	9.0341
n4	306.1668	305.7141	0.4527	−24.8596	3.4417
n5	310.2255	309.8248	0.4006	−24.9168	3.9724
n6	316.2202	315.7490	0.4711	−24.9999	3.2686
n7	307.4993	306.5631	0.9363	−24.8784	0.2861
n8	314.9743	313.8540	1.1203	−24.9828	−0.4935
n9	317.7783	316.7337	1.0446	−25.0212	−0.1894

**Table 7:** Results of battery temperatures for forced convection.

Run	Tmax	Tmin	dT	Tmax_SN	dT_SN
f1	303.1744	303.0907	0.0837	−24.8169	10.7724
f2	308.1296	308.0619	0.0677	−24.8873	11.6957
f3	313.1010	313.0450	0.0561	−24.9568	12.5132
f4	303.3960	303.1932	0.2029	−24.8201	6.9279
f5	308.3106	308.1415	0.1691	−24.8899	7.7196
f6	313.4262	313.2312	0.1950	−24.9614	7.1005
f7	303.5501	303.2613	0.2888	−24.8223	5.3946
f8	308.7654	308.4337	0.3317	−24.8963	4.7920
f9	313.5813	313.3024	0.2790	−24.9635	5.5447

**Table 8:** Response Table for maximal battery temperature for natural convection.

	C-rate	FST	HTC
Level 1	−24.9004	−24.8585	−24.9401
Level 2	−24.9254	−24.9328	−24.9265
Level 3	−24.9608	−24.9953	−24.9200

Delta	0.0604	0.1368	0.0200
Rank	2	1	3
Contribution	28	63	9

**Table 9:** Response Table for maximal battery temperature for forced convection.

	C-rate	FST	HTC
Level 1	−24.887	−24.8198	−24.8915
Level 2	−24.8904	−24.8912	−24.8903
Level 3	−24.894	−24.9606	−24.8897

Delta	0.0070	0.1408	0.0018
Rank	2	1	3
Contribution	5	94	1

**Table 10:** Response Table for temperature uniformity for natural convection.

	C-rate	FST	HTC
Level 1	8.4189	3.8402	3.5226
Level 2	3.5609	3.9696	3.8941
Level 3	−0.1323	4.0378	4.4309

Delta	8.5512	0.1976	0.9083
Rank	1	3	2
Contribution	89	2	9

**Table 11:** Response Table for temperature uniformity for forced convection.

	C-rate	FST	HTC
Level 1	11.6604	7.6983	7.5550
Level 2	7.2494	8.0691	8.0561
Level 3	5.2438	8.3861	8.5425

Delta	6.4167	0.6878	0.9875
Rank	1	3	2
Contribution	79	9	12

## References

[1] Yu M, Bai B, Xiong S, Liao X (2021). Evaluating environmental impacts and economic performance of remanufacturing electric vehicle lithium-ion batteries. Journal of Cleaner Production.

[2] Spitthoff L, Wahl MS, Vie PJ, Burheim OS (2023). Thermal transport in lithium-ion batteries: The effect of degradation. Journal of Power Sources.

[3] Tian J, Liu X, Li S, Wei Z, Zhang X (2023). Lithium-ion battery health estimation with real-world data for electric vehicles. Energy.

[4] Lopez FA, Billy RG, Müller DB (2023). Evaluating strategies for managing resource use in lithium-ion batteries for electric vehicles using the global MATILDA model. Resources, Conservation and Recycling.

[5] Vashisht S, Rakshit D, Panchal S, Fowler M, Fraser R (2023). Thermal behaviour of Li-ion battery: An improved electrothermal model considering the effects of depth of discharge and temperature. Journal of Energy Storage.

[6] Farulla GA, Palomba V, Alosio D, Brunaccini G, Ferraro M (2023). Optimal design of lithium ion battery thermal management systems based on phase change material at high current and high environmental temperature. Thermal Science and Engineering Progress.

[7] He P, Lu H, Fan Y, Ruan H, Wang C (2023). Numerical investigation on a lithium-ion battery thermal management system utilizing a double-layered I-shaped channel liquid cooling plate exchanger. International Journal of Thermal Sciences.

[8] Hasan HA, Togun H, Abed AM, Mohammed HI, Biswas N (2023). A novel air-cooled Li-ion battery (LIB) array thermal management system–a numerical analysis. International Journal of Thermal Sciences.

[9] Celen A, Kalkan O (2021). Numerical investigation on the usage of finned surface in lithium nickel manganese cobalt oxides batteries by using air cooling method. Energy Storage.

[10] Fan Z, Fu Y, Liang H, Gao R, Liu S (2023). A module-level charging optimization method of lithium-ion battery considering temperature gradient effect of liquid cooling and charging time. Energy.

[11] Wankhede S, Kamble L (2023). Performance investigation of electric vehicle battery thermal management system using nano fluids as coolants on ANSYS CFX software. Energy Storage.

[12] Kermani JR, Taheri MM, Shafii MB, Moosavi A (2023). Analytical solution, optimization and design of a phase change cooling pack for cylindrical lithium-ion batteries. Applied Thermal Engineering.

[13] Patel JR, Rathod MK (2023). Novel approach for the performance augmentation of phase change material integrated battery thermal management system for number of charging/discharging cycles. Energy Storage.

[14] Hai T, Abidi A, Abed AM, Zhou J, Malekshah EH (2022). Three-dimensional numerical study of the effect of an air-cooled system on thermal management of a cylindrical lithium-ion battery pack with two different arrangements of battery cells. Journal of Power Sources.

[15] Hasan HA, Togun H, Abed AM, Biswas N, Mohammed HI (2023). Thermal performance assessment for an array of cylindrical Lithium-Ion battery cells using an Air-Cooling system. Applied Energy.

[16] Chen S, Zhang G, Zhu J, Feng X, Wei X (2022). Multi-objective optimization design and experimental investigation for a parallel liquid cooling-based Lithium-ion battery module under fast charging. Applied Thermal Engineering.

[17] Xu J, Chen Z, Qin J, Minqiang P (2022). A lightweight and low-cost liquid-cooled thermal management solution for high energy density prismatic lithium-ion battery packs. Applied Thermal Engineering.

[18] Li J, Tang A, Shao X, Jin Y, Chen W (2022). Experimental evaluation of heat conduction enhancement and lithium-ion battery cooling performance based on h-BN-based composite phase change materials. International Journal of Heat and Mass Transfer.

[19] Zhou Z, Wang D, Peng Y, Li M, Wang B (2022). Experimental study on the thermal management performance of phase change material module for the large format prismatic lithium-ion battery. Energy.

[20] Zhang H, Li C, Zhang R, Lin Y, Fang H (2020). Thermal analysis of a 6s4p Lithium-ion battery pack cooled by cold plates based on a multi-domain modeling framework. Applied Thermal Engineering.

[21] Ceyda K, Alkaya A (2020). Investigation of Thermal Behavior of Lithium-Ion Batteries under Different Loads. European Mechanical Science.

[22] Celik A, Coban H, Göcmen S, Ezan MA, Gören A (2019). Passive thermal management of the lithium-ion battery unit for a solar racing car. International Journal of Energy Research.

[23] Paccha Herrera E, Calderón Muñoz WR, Orchard M, Jaramillo F, Medjaher K (2020). Thermal modeling approaches for a LiCoO2 lithium-ion battery—A comparative study with experimental validation. Batteries.

[24] Kumar RS, Jithin K, Rajesh P (2023). Lithium-ion ferrous phosphate prismatic cell aging analysis and assessment for the development of battery management systems. Journal of Energy Storage.

[25] Kim US, Yi J, Shin CB, Han T, Park S (2011). Modeling the dependence of the discharge behavior of a lithium-ion battery on the environmental temperature. Journal of The Electrochemical Society.

[26] Kwon KHK, Shin CB, Kang TH, Kim CS (2006). A two-dimensional modeling of a lithium-polymer battery. Journal of Power Sources.

[27] Kim US, Shin CB, Kim CS (2009). Modeling for the scale-up of a lithium-ion polymer battery. Journal of Power Sources.

